# Novel pharmacotherapeutic treatments for cocaine addiction

**DOI:** 10.1186/1741-7015-9-119

**Published:** 2011-11-03

**Authors:** Daryl Shorter, Thomas R Kosten

**Affiliations:** 1 Menninger Department of Psychiatry and Behavioral Science, Baylor College of Medicine, 1977 Butler Blvd., Suite E4.400, Houston, TX 77030, USA; 2 MEDVAMC - 2002 Holcombe Blvd., Bldg 110, Room 229, Houston, TX 77030, USA

## Abstract

Cocaine is a stimulant that leads to the rapid accumulation of catecholamines and serotonin in the brain due to prevention of their re-uptake into the neuron that released the neurotransmitter. Cocaine dependence is a public health concern and cause of significant morbidity and mortality worldwide. At present, there are no approved medications for the treatment of this devastating illness, and behavioral interventions have proven to be of limited use. However, there have been a number of recent trials testing promising agents including dopamine agonists, GABAergic medications and the cocaine vaccine. Here we discuss the most recent human clinical trials of potential medications for treatment of cocaine dependence, as well as pre-clinical studies for another promising agent, levo tetrahydropalmatine. Examination of these recent findings shows promise for GABAergic medications and the cocaine vaccine, as well as unique medications such as disulfiram, whose mechanism remains to be determined. Future work may also confirm specific subgroups of patients for treatment response based on clinical characteristics, biomarkers and pharmacogenetics. This review highlights the need for further, bigger studies in order to determine optimal clinical usage.

## Introduction

Cocaine is a stimulant that leads to the rapid accumulation of catecholamines and serotonin in the brain due to prevention of their re-uptake into the neuron that released the neurotransmitter. Cocaine use disorders are widely accepted as a significant cause of morbidity and mortality. Cocaine use is associated with numerous acute and chronic medical complications, ranging from coronary syndromes, myocardial infarction, and respiratory disease to neurologic and psychiatric consequences such as cerebral hemorrhage, mood disorders, and psychosis [1,2]. Additionally, cocaine use has been associated with increased risk of HIV, hepatitis B and C, and violence [3-6].

Development of effective treatments for cocaine dependence is necessary to reduce the impact of this illness upon both the individual and society. These effective treatments need most importantly to reduce cocaine use and to have excellent compliance, which has encouraged depot and other long lasting formulations. Currently, however, there are no US Food and Drug Administration (FDA) approved medications for the treatment of this illness, and behavioral therapies alone have demonstrated limited efficacy [7]. Our growing understanding of cocaine neurobiology has translated into numerous studies of pharmacologic agents for treatment of cocaine dependence in both animal and human models. These models include human laboratory drug administration studies using surrogate endpoints such as craving, subjective effects, and behavioral choices of money versus drug. This article reviews findings from recent cocaine pharmacotherapy clinical trials in humans that target dopamine and gamma-aminobutyric acid (GABA) neurotransmitter systems or uniquely target the cocaine itself through a vaccine preventing cocaine from getting into the brain. Additionally, preclinical studies for a novel medication, levo-tetrahydropalmatine (*l*-THP) are discussed.

### Dopamine agonists

The final common pathway for reward and reinforcement associated with substances of abuse has been extensively shown to result from dopamine release from the ventral tegmental area (VTA) to the nucleus accumbens (NAc), prefrontal cortex (PFC), as well as other structures [8]. The subjective effects and euphoria of cocaine use are attributed to blockade of the dopamine transporter, reuptake inhibition, and increased levels of extracellular dopamine in the mesolimbic and mesocortical pathways. Chronic use of cocaine is associated with relative dopaminergic hypofunctioning and may underlie the withdrawal symptoms and craving observed in cocaine-dependent persons who have recently established abstinence [9]. As a result, dopamine agonists have been studied as potential pharmacotherapeutic options, since they serve to offset neuroadaptive changes associated with chronic use such as decreased dopamine D2 receptor binding [10] and have been used successfully for the treatment of both opiate and nicotine dependence, highlighting their potential utility for treatment of other forms of substance abuse [11,12]. Recent trials for dopamine agonists have mainly focused on amphetamine derivatives such as dextroamphetamine (d-amphetamine) and methamphetamine, as well as modafinil and disulfiram.

D-amphetamine has shown mixed results in regards to its ability to attenuate subjective effects [13,14]. In clinical study oral methamphetamine demonstrated an ability to reduce both craving and cocaine use, as evidenced by a statistically significant increase in the percentage of cocaine-negative urines [15]. Concerns regarding the addictive liability of these agents in persons with a history of substance dependence remain a consideration, particularly since the dependence syndrome (characterized by development of tolerance, withdrawal, and inability to control use) has been clearly established for amphetamines [16]. Overall, slow release formulations with strong diversion resistant formulations would be essential for use of amphetamine as a treatment agent. So far, no such formulation has been developed although a lysine conjugate of amphetamine that has been marketed for treating childhood attention deficit disorder shows some promise. However, these medications have shown some promise and merit further clinical investigation, particularly as cognitive enhancement during early abstinence becomes a more prominent focus in addictions research and these agents may also be helpful in this area.

Modafinil is a novel stimulant-like medication that promotes wakefulness and serves as a treatment for hypersomnia and narcolepsy. The mechanism of action is only partially understood at this time; however, there is evidence that this agent acts as a central alpha-1 adrenergic agonist [17], reduces basal cerebral GABA activity [18], and increases both dopamine and glutamate release in select areas of the brain [19,20]. Chronic cocaine use results in a hypodopaminergic state as well as depletion of both extracellular glutamate levels and glutamatergic synaptic strength in the nucleus accumbens [21]. These neurotransmitter systems serve as the principal targets of modafinil's effects and promising results in human clinical trials for treatment of cocaine dependence have been observed.

The safety of co-administration of modafinil and cocaine was established in a randomized, double blind, placebo-controlled study (N = 10) conducted by Dackis *et al*. [22]. Modafinil demonstrated no significant exacerbating effects on vital sign measures or electrocardiogram (EKG) findings. As a secondary measure, pretreatment with modafinil at two oral dosages (200 mg and 400 mg, respectively) demonstrated an ability to significantly attenuate euphoria from intravenously administered cocaine in one subjective measure (*P *= 0.02). Importantly, this early study suggested safety as well as the possibility of a cocaine-blunting effect with modafinil.

In addition to examining the impact of modafinil on the euphorigenic effects of cocaine, early research focused on the similarity of modafinil to stimulant medications in order to assess the extent of its potential abuse liability in both humans and animals [23,24]. Overall, the perceived risk of abuse associated with this medication has been found to be limited, since modafinil possesses, at most, a weakly reinforcing effect [24]. More recently, a double-blind, randomized, outpatient study (N = 12) conducted in cocaine addicted subjects found that modafinil at three different doses (200 mg, 400 mg, and 600 mg/day, respectively) failed to elicit a reinforcing effect, as the medication was chosen at the same frequency as placebo and was not associated with positive subjective effects [25].

Recent clinical trials of modafinil in human subjects have highlighted either (1) the medication's impact upon actual cocaine use or (2) its ability to mitigate symptoms associated with cocaine abstinence. The rationale for using this medication relates to its stimulant like properties and potential efficacy as a substitution agent that can reduce craving for cocaine. With regard to its effect upon cocaine use, modafinil was examined in a randomized, double-blind, placebo-controlled study (N = 210) at two doses (200 mg and 400 mg); however, no significant differences were observed between the modafinil and placebo groups in their change in the average weekly percentage of cocaine non-use days [26]. Of note, modafinil 200 mg demonstrated a significant reduction in craving as a secondary outcome. Interestingly, post-hoc analysis also found an increase in cocaine non-use days among those with co-morbid alcohol dependence who were treated with modafinil, suggesting a possible target subgroup among cocaine-addicted persons.

The impact of modafinil on sleep in chronic cocaine users was examined in a randomized, placebo-controlled, inpatient study (N = 20) by Morgan *et al*. The researchers found that modafanil 400 mg decreased nighttime sleep latency and increased slow-wave sleep time [27]. Additionally, by the third week of abstinence from cocaine, the modafinil group experienced longer total sleep time and shorter rapid eye movement (REM) latency. Ultimately, modafanil demonstrated a normalizing effect on sleep, which could be particularly important during early phases of abstinence since sleep disturbance has been associated with relapse into substance abuse and there are currently no proven pharmacologic treatment options for this form of insomnia [28]. Modafinil continues to represent an area of exciting promise in the pharmacologic management of cocaine dependence, since it appears to have limited reinforcing effects, reduces cocaine craving, decreases cocaine use among sub-populations of cocaine users (that is, those with co-morbid alcohol dependence), and treats symptoms characteristic of the abstinence syndrome.

Disulfiram, an aldehyde dehydrogenase inhibitor as well as a dopamine-beta-hydroxylase (DBH) inhibitor, is approved for treatment of alcohol dependence. Because the DBH enzyme converts dopamine to norepinephrine, its inhibition leads to a decrease in neuronal and synaptic norepinephrine levels relative to dopamine [29]. This neurobiochemical mechanism has been proposed as a potential therapy for cocaine dependence, extensively studied in human clinical trials, and found to result in modulation of the reinforcing properties of cocaine and reduction of cocaine use [30-32]. Most recently, a double-blind, placebo-controlled randomized clinical trial (RCT) of disulfiram for treatment of cocaine dependence in methadone-stabilized individuals showed that in the group receiving 250 mg/day, there was a significant decrease in cocaine-positive urines over time when compared to lower doses of the medication or placebo [33]. Interestingly, lower dosages of the medication (62.5 mg and 125 mg, respectively) were associated with increased self-report of cocaine use as well as cocaine-positive urines. Future study of disulfiram should focus on dosing strategy as well as identification of subpopulations in which the drug is maximally effective.

Nepicastat, a selective DBH-inhibitor that has yet to come to market, is currently under investigation for treatment of cocaine dependence. In preclinical trials, nepicastat has demonstrated an ability to (1) increase synaptic levels of dopamine, (2) decrease brain norepinephrine levels, and (3) block cocaine-induced reinstatement of cocaine seeking in rats without affecting food-primed reinstatement of food seeking [34]. These results suggest similarity between disulfiram and nepicastat in terms of their ability to attenuate responses to drug-related cues and represent an exciting prospect in the pharmacotherapy of cocaine dependence.

### Serotonergic agents

In addition to its action at the dopamine transporter, cocaine binds to the norepinephrine transporter (NET) and serotonin transporter (SERT), causing inhibition of presynaptic uptake of these monoamines as well [35]. During acute cocaine intoxication, enhanced dopamine transmission in the nucleus accumbens is accompanied by increased release of serotonin [36], and there is evidence supporting the contributing role of serotonin to cocaine reward and reinforcement [37]. In the dorsal raphe nucleus, increased extracellular levels of serotonin result in activation of 5-hydroxytryptamine-1a (5HT-1a) autoreceptors and reduce firing of these neurons [38]. Cocaine withdrawal is characterized by serotonin depletion throughout the brain and decreased levels of 5HT in the nucleus accumbens [39]. Interestingly, in rat studies, enhancement of serotonergic transmission in the nucleus accumbens through administration of exogenous 5HT served to offset the dopamine deficit caused by cocaine withdrawal [40].

Given these interactions, preclinical studies have examined the impact of pharmacologic manipulation of the serotonin system on cocaine effects. Early studies in rats demonstrated that serotonin-enhancing medications were associated with decreased self-administration of cocaine [41,42]. However, human clinical trials examining the efficacy of serotonergic medications (that is, selective serotonin reuptake inhibitors (SSRI)) in the treatment of cocaine dependence have yielded mixed results [43-46].

The discrepancy between findings from animal and human trials regarding the effect of serotonergic medications might be due to differences in the circumstances leading to reinstatement of cocaine use. In a review by Filip *et al*., the authors stress that activity at different subtypes of serotonin receptors can have different effects on cocaine use. For example, cocaine-seeking behavior induced by environmental cues (conditioned stimuli) can be modified by serotonergic medications that counteract the 5HT deficit of withdrawal or suppress cocaine-induced changes in this neurotransmitter system [47].

Moeller *et al*. tested this particular hypothesis in a recent double-blind, placebo-controlled RCT (N = 76) examining citalopram (20 mg/day) combined with cognitive behavioral therapy (CBT) and contingency management (CM) over 12 weeks in the treatment of cocaine dependence [48]. The subjects in the citalopram group demonstrated a substantial decrease in the number and probability of cocaine positive urine drug screens. As a future direction, the use of behavioral therapy platforms to address response to drug-related cues in conjunction with serotonergic agents may be the necessary adjunct to treatment to improve the efficacy of these medications.

Ibogaine, the primary indole alkaloid found in the root bark of the African shrub, *Tabernanthe iboga*, has shown promise not only in the treatment of cocaine dependence, but in alcohol, opiate, and methamphetamine dependence as well, representing the first agent that could be beneficial in multiple substance use disorders (SUDs) [49]. The pharmacologic properties of ibogaine have been extensively studied and ibogaine has demonstrated an affinity for a number of receptor sites including opioid (kappa, mu, and delta), N-methyl-d-aspartate (NMDA), sigma (1 and 2), dopamine transporter (DAT), SERT, and nicotinic [50]. The action of this medication at multiple receptor sites in combination has been identified as the predominant factor underlying the putative anti-addictive properties of ibogaine. However, despite general agreement on the sites of activity of this medication, there remain some contradictory findings in regards to the action of ibogaine within the brain. For example, Baumann *et al *found that ibogaine and noribogaine had little effect on extracellular levels of dopamine in the rat nucleus accumbens, while Glick *et al*. showed that these agents caused a significant decrease in dopamine levels [51,52]. These differences may be due to differences in study design, method of administration of ibogaine and/or the gender of the animals. In regards to serotonergic transmission, both ibogaine and noribogaine have been shown to increase extracellular 5HT in the brain [52].

Regarding its impact on cocaine use, both ibogaine and its active metabolite, noribogaine, have been shown to significantly decrease cocaine self-administration, an effect that persisted in some animals for several days following only a single dose [53]. Although ibogaine is associated with potentially intolerable side effects, such as tremor and impaired coordination, noribogaine does not appear to cause these problems, suggesting this agent may be easier to tolerate [51].

One focus in clinical trials has been to isolate specific iboga alkaloids, such as 18-methoxycoronaridine (18-MC), in order to test their efficacy in treating chemical dependency with minimal adverse reactions [54]. Importantly, 18-MC has demonstrated an ability to reduce cocaine self administration without apparent toxicity [55]. Of note, 18-MC has also shown an ability to reduce self administration of other drugs of abuse, including morphine, methamphetamine, nicotine, and alcohol [54]. To date, there have been no human clinical trials for treatment of cocaine dependence with iboga alkaloids; however, this class of medications, with enhanced safety profiles, could represent an exciting intervention in the treatment of not only cocaine dependence, but other drug use disorders as well.

### GABA-ergic medications

There is significant evidence for the involvement of brain GABA systems in perpetuation of the addictive process and the enhancement of GABA activity in addicted individuals is associated with decrease in drug craving and relapse [56]. More specifically, GABA has demonstrated the ability to suppress dopamine release in the striatum, also blunting cocaine-induced release of dopamine in animals. The translation of our understanding of this neurobiology into successful human clinical trials has been somewhat difficult. An additional complicating factor with this class of medications is that given the widespread distribution of the GABAergic system within the central nervous system (CNS), these medications may be associated with various side effects. Recent studies of vigabatrin, baclofen, valproate, and topiramate yield mixed results in their ability to improve outcomes in cocaine-addicted persons.

Vigabatrin, also known as gamma-vinyl-GABA (GVG), is an irreversible inhibitor of GABA transaminase that reduces the breakdown of GABA, thereby increasing its activity within the synapse [56]. In preclinical studies GVG has been shown to reduce cocaine-induced dopamine release by 25% or more in laboratory animals; however, there has also been an association with visual field defects in 1/3 of persons exposed to the medication for extended periods [57]. In a recently published RCT (N = 103), GVG, when compared to placebo, resulted in a higher percentage of subjects achieving and maintaining abstinence from cocaine by the end of the trial (20% of GVG group (n = 50) versus 7.5% of placebo (n = 53)) [58]. Interestingly, participants in the GVG group were also more likely to report abstinence from alcohol by the end of study (43.5% versus 6.3%). Study retention was significantly higher in the GVG group and the medication was well tolerated. Ongoing study of GVG is necessary, particularly given its potential implications for the subpopulation of patients with cocaine dependence and comorbid alcohol abuse/dependence.

Baclofen, a GABA(B) receptor agonist, is used widely as a treatment of spasticity and has demonstrated efficacy in preclinical trials for treatment of cocaine dependence. In various rat studies, baclofen demonstrated the ability to reduce cocaine self-administration [59,60] and cocaine-induced reinstatement [61], cocaine-seeking behaviors [62], and cocaine-induced release of dopamine (DA) in the shell of the nucleus accumbens [63].

In an earlier double blind, placebo-controlled RCT (N = 70), Shoptaw *et al*. found that administration of baclofen (20 mg three times a day) resulted in statistically significant reductions in cocaine use when compared to placebo [64]. Although baclofen did not demonstrate a statistically significant impact on cocaine craving, participants were more likely to submit cocaine-negative urine samples between weeks three to eight of treatment. This finding may suggest the possible utility of this medication in those needing assistance with relapse prevention, rather than abstinence initiation. Of note, the authors also examined the impact of the level of cocaine use at baseline on treatment outcome, finding that those with a more severe form of cocaine dependence were more likely to respond to baclofen treatment. A more recent multisite, double-blind RCT assessed the safety and efficacy of 60 mg baclofen treatment for in 160 subjects diagnosed with severe cocaine dependence [65]. The groups (treatment versus placebo) did not differ in terms of treatment retention rates or change in mean weekly percentage of cocaine non-use days. The limited success of baclofen may be due to its use in a population identified as severely cocaine dependent or because it was used to assist with abstinence initiation, rather than relapse prevention. Examination of baclofen in subjects with mild to moderate cocaine dependence or in those who have already established abstinence may yield more promising results.

Valproate, which enhances GABA levels by increasing glutamic acid decarboxylase (GAD) activity and inhibiting GABA transaminase activity, was examined in a RCT, within-subjects, crossover study design meant to assess its effect on cue-induced cocaine craving [66]. Subjects identified as crack cocaine dependent (N = 20) were titrated to 1, 500 mg/day of valproate and subsequently exposed to a series of neutral and cocaine-related cues. Interestingly, under treatment conditions with valproate, participants reported higher craving (that is, 'desire to use now') in response to cue exposure when compared to placebo condition. Due to limited sample size, however, further study of this medication may still be warranted.

Tiagabine, a GABA reuptake inhibitor, has been examined in two recent human clinical trials. In an earlier RCT (N = 141), the group receiving tiagabine (20 mg/day) did not differ significantly from placebo in terms of cocaine craving and global function [67]. Additionally, there was no significant change in cocaine use in either the study or placebo groups. A later study compared the impact of tiagabine versus lorazepam, a benzodiazepine and GABA-enhancing medication, and placebo. The authors found that tiagabine increased slow-wave sleep by three times in those recently establishing abstinence from cocaine use [68]. Importantly, tiagabine did not differ from placebo in regards to impact on cognitive function (that is, vigilance task, measures of impulsivity), while lorazepam was found to cause next-day impairment. There may be a possible indication for tiagabine in the period of early abstinence, since this medication might improve the sleep disturbance characteristic of withdrawal; however, additional study is warranted to determine the extent of its effect upon establishment of abstinence, cocaine craving, and relapse.

The final GABAergic medication to have been recently tested in human clinical study is topiramate which, in addition to potentiation of GABA(A) receptor mediated input, antagonizes glutamatergic afferents to the mesocorticolimbic dopaminergic system [69]. Kampman *et al*. demonstrated the efficacy of topiramate in the treatment of cocaine dependence in a randomized, double-blind, placebo-controlled RCT (N = 40) [70]. Over the first eight weeks of the study, topiramate was titrated by 25 mg/week to a target dose of 200 mg/day. During that period, topiramate did not demonstrate a statistically significant ability to reduce cocaine use. After week eight, topiramate-treated subjects were more likely to be abstinent from cocaine when compared to placebo, as measured by twice-weekly urine benzoylecgonine test (UBT).

In an open label, outpatient study of cocaine dependent men (N = 28), participants received topiramate, ranging in dose from 25 to 300 mg/day [71]. Interestingly, the only statistically significant finding of the study was a decrease in craving intensity, although this effect was seen in only 25% of participants. Further study of topiramate in a larger, placebo-controlled RCT is necessary in order to determine the extent of this medication's impact upon craving. Also, the addition of genetic analysis into future trials might help to determine underlying differences in subgroups of patients and provide clues regarding differential response patterns.

### Levo-tetrahydropalmatine

Levo-tetrahydropalmatine (*l*-THP), a tetrahydroprotoberberine alkaloid, is one of the primary active agents found in the plant genera *Corydalis *and *Stephania *[72]. Two species in particular, *Corydalis ambiguo *and *Stephania tetranda*, are included among the 50 fundamental herbs in Chinese traditional medicine and are used for various purposes including treatment of anxious insomnia and chronic pain due to their sedative/hypnotic and analgesic properties, respectively [72,73]. The mechanism of action of *l*-THP, elucidated by studies in rats, centers around antagonism of dopamine D1 and D2 receptors [74] and was further evidenced by its ability to reverse the effects of apomorphine, a known dopamine receptor agonist [75]. Additionally, there is evidence to suggest antagonist activity at D3 receptor sites [72]. In addition to activity in the dopaminergic system, *l*-THP has shown an ability to act as both an alpha-1 adrenergic receptor antagonist (Mantsch, 2007) and an allosteric modulator of gamma-aminobutyric acid (GABA)_A _receptors [76]. Interestingly, this agent has a similar effect to modafinil, but acts through a different mechanism.

Taken altogether, the unique neurobiochemical profile of *l*-THP may translate into an exciting area of promise in the study of pharmacotherapy for cocaine dependence. In rats, *l*-THP has demonstrated an ability to dose-dependently reduce cocaine self-administration and attenuate cocaine-induced reinstatement under both fixed-ratio [72] and progressive-ratio scheduling [77]. Further, oral administration of *l*-THP was found to attenuate cocaine-seeking behavior within various reinstatement paradigms (that is, cocaine, stress, and environmental associated cues) [78]. These findings in animal studies suggest that *l*-THP may represent an effective future pharmacotherapeutic option in the treatment of cocaine dependence.

### Cocaine vaccine

Substance abuse vaccines represent an exciting area of promise in the treatment of chemical dependency. The introduction of these agents into our pharmacologic armamentarium represents an important shift in our conceptualization of drug use, since its foundation rests upon the idea of substances of abuse as agents 'foreign' to the body and susceptible to immunologic mechanisms. Currently, clinical trials for vaccines treating both cocaine and nicotine dependence are ongoing, with vaccines for methamphetamine and heroin in preclinical development stages.

The cocaine vaccine, TA-CD, is composed of a cocaine hapten conjugated to inactivated cholera toxin B, resulting in the creation of a molecule capable of stimulating an antibody response [79]. These antibodies are cocaine specific; ingestion of the substance by any means (intranasal, inhalational, intravenous) results in its binding and the creation of immune complexes unable to cross the blood-brain-barrier due to their relatively larger size. These molecules are then broken down by cholinesterases in the circulation, converting the cocaine into inactive metabolites that are then excreted [80].

In phase I clinical study (N = 34), participants receiving TA-CD were able to mount an immunologic response resulting in the creation of cocaine-specific antibodies [81]. Subjects reported a reduction in the subjective effects and euphoria from smoked cocaine [82,83].

Similarly positive findings were found during a phase II clinical study of two dose levels (100 ug × 4 injections, or 400 ug × 5 injections)[82]. Subjects receiving the higher dose were found to have higher mean antibody levels and were also more likely to remain abstinent at six-month follow-up (relapse in 89% in the low-dose group compared to 43% in the high-dose group) [82,80].

In the initial phase IIb trial (N = 115), TA-CD was administered to methadone-maintained cocaine-dependent individuals at a single dose (360 ug × 5 injections) in comparison to placebo. Those subjects with high antibody production were found to have a greater percentage of cocaine-free urines [84]. In all phases of testing, the safety profile of the vaccine was favorable, with any serious adverse effects being deemed unrelated to the vaccine. Currently, TA-CD is undergoing large-scale, multisite, phase IIb clinical testing, although there are limitations in these studies including only 40% of the patients attaining fully blocking levels of antibodies. Better adjuvants are clearly needed.

### Summary/Future directions of research

These prospects in the pharmacologic management of cocaine dependence have demonstrated in human clinical trials an ability to reduce subjective reward, craving, and withdrawal symptoms associated with cocaine use, however, there is still much progress to be made, before they are viable widespread treatments Of the dopamine agonist medications, although they have been demonstrated to decrease the euphoric, as well as, in some cases, the withdrawal symptoms, concerns regarding the addictive liability of amphetamine-type medications continue to limit the widespread acceptance and use of this treatment. Further, findings in clinical trials of GABAergic medications have been less clear in their demonstration of significant efficacy in treating cocaine dependence. They have shown some ability to reduce cocaine craving or to improve sleep duration and quality in those recently abstaining from cocaine, and cocaine abusers with comorbid alcohol dependence may represent a subpopulation particularly responsive to the effects of GVG. The cocaine vaccine, TA-CD, has demonstrated the ability to elicit an immunologic response capable of reducing the subjective reward of cocaine use in both animals and humans. These results make immunologic treatment of substance use disorders an exciting direction for treatment of not only cocaine dependence, but other substances as well. Finally, findings from preclinical trials for *l*-THP suggest this medication, already used for centuries as an herbal medication for other disorders, may be helpful in reducing cocaine use.

## Conclusions

Examination of these recent findings shows promise for GABAergic medications and the cocaine vaccine, as well as for unique medications such as disulfiram, whose mechanism remains to be determined. Further studies with all of these agents are likely to be worthwhile, although the focus for disulfiram needs to be on its potential mechanism of action such as its inhibition of dopamine beta hydroxylase using more specific agents like nepicastat. Alternatively, the copper chelation caused by disulfiram is being examined using more specific copper chelating compounds that have been developed for Wilson's disease. Alternative agents with fewer side effects are being examined for vigabitrin. Finally, more effective vaccines have been developed in animals using better adjuvants than alum such as squalene or MPL60 as alternatives. Clearly progress has been made in developing new and unique agents and mechanisms of action for reducing cocaine dependence. Specific barriers to developing better treatments are clearly related to the overall challenges of getting industry support and FDA approval when no previous medication has been approved for cocaine dependence. Small companies are working well with the US National Institute on Drug Abuse (NIDA) to develop these therapies, but the larger pharmaceutical industry will be essential partners for the FDA process in bringing any of these treatments to market. Some aspects of cocaine addiction need to be better understood in order to make further progress. In particular, the mechanisms of action relevant to disulfiram's efficacy for cocaine need to be identified in order to develop better, safer, and more specific agents. Furthermore, it is likely that treatment will need to be more tailored for specific subsets of patients. These subsets may be identified through clinical characteristics (severity of dependence based on number of days of month of using cocaine), biomarkers (urine levels of the cocaine metabolite benzoylecognine or having immunoglobulin M (IgM) antibodies to cocaine before first vaccine dose) and pharmacogenetic markers

(functional polymorphisms associated with the gene coding for dopamine beta hydroxylase),

## List of abbreviations

CBT: cognitive behavioral therapy; CM: contingency management; CNS: central nervous system; d-amphetamine: dextroamphetamine; DA: dopamine; DAT: dopamine transporter; DBH: dopamine-beta-hydroxylase; EKG: electrocardiogram; FDA: US Food and Drug Administration; GABA: gamma-aminobutyric acid; GVG: gamma-vinyl-GABA; IgM: immunoglobulin M; *l*-THP: levo-tetrahydropalmatine; NAc: nucleus accumbens; NET: norepinephrine transporter; NMDA: N-methyl-d-aspartate; PFC: prefrontal cortex; RCT: randomized clinical trial; REM: rapid eye movement; SERT: serotonin transporter; SSRI: selective serotonin reuptake inhibitors; SUDs: substance use disorders; UBT: urine benzoylecgonine test; VTA: ventral tegmental area; 5HT-1a: 5-hydroxytryptamine-1a; 18-MC: 18-methoxycoronaridine

## Competing interests

Dr. Kosten has served as a consultant to Alkermes, Biotie, Reckitt Benkizer, Catalyst, Titan, NABI, Teva, Lundbeck, Pfizer, and GSK. Dr. Shorter has no competing interests.

## Authors' contributions

Both authors contributed equally to the writing and have read and approved the final manuscript.

## Pre-publication history

The pre-publication history for this paper can be accessed here:

http://www.biomedcentral.com/1741-7015/9/119/prepub
